# Evaluating the Learning Procedure of CNNs through a Sequence of Prognostic Tests Utilising Information Theoretical Measures

**DOI:** 10.3390/e24010067

**Published:** 2021-12-30

**Authors:** Xiyu Shi, Varuna De-Silva, Yusuf Aslan, Erhan Ekmekcioglu, Ahmet Kondoz

**Affiliations:** Institute for Digital Technologies, Loughborough University London, Queen Elizabeth Olympic Park, Here East, London E20 3BS, UK; X.Shi@lboro.ac.uk (X.S.); yusufaslan093@gmail.com (Y.A.); e.ekmekcioglu@lboro.ac.uk (E.E.); A.Kondoz@lboro.ac.uk (A.K.)

**Keywords:** convolutional neural networks, learning procedure, information theory, mutual information

## Abstract

Deep learning has proven to be an important element of modern data processing technology, which has found its application in many areas such as multimodal sensor data processing and understanding, data generation and anomaly detection. While the use of deep learning is booming in many real-world tasks, the internal processes of how it draws results is still uncertain. Understanding the data processing pathways within a deep neural network is important for transparency and better resource utilisation. In this paper, a method utilising information theoretic measures is used to reveal the typical learning patterns of convolutional neural networks, which are commonly used for image processing tasks. For this purpose, training samples, true labels and estimated labels are considered to be random variables. The mutual information and conditional entropy between these variables are then studied using information theoretical measures. This paper shows that more convolutional layers in the network improve its learning and unnecessarily higher numbers of convolutional layers do not improve the learning any further. The number of convolutional layers that need to be added to a neural network to gain the desired learning level can be determined with the help of theoretic information quantities including entropy, inequality and mutual information among the inputs to the network. The kernel size of convolutional layers only affects the learning speed of the network. This study also shows that where the dropout layer is applied to has no significant effects on the learning of networks with a lower dropout rate, and it is better placed immediately after the last convolutional layer with higher dropout rates.

## 1. Introduction

The fields of Artificial Intelligence (AI) and Machine Learning (ML) have been developing rapidly over recent years. The success, especially in deep learning during the last decade, has been rapid with unpredictable achievements in international challenges. Deep learning algorithms have made remarkable progress on numerous ML tasks and dramatically improved the state-of-the-art in many functional areas ranging from visual image recognition to understanding languages from audio [1,2,3,4,5]. As a result of this success, deep learning models have been used in various application areas such as criminal justice, medicine and finance [6].

Despite these successes, there is still no comprehensive understanding of the optimisation process or the internal organisation of deep neural networks, and they are often criticised for being used as mysterious “black boxes” [7]. Deep learning models usually contain millions of parameters and functions. Humans cannot understand this representation and the relations of the parameters, and also cannot physically interpret the results of models. This lack of understanding can lead to a belief that the models are complex, mysterious and untrustworthy. Additionally, there is no way to know if the reasons behind the results are ill-formatted, biased or even wrong, which can raise many ethical, financial and legal issues. Studies on Explainable Machine Learning (EML) are currently attempting to provide explanations and solutions to these kinds of problems by giving the reasoning behind them. 

Convolutional Neural Networks (CNNs) are widely used as models for a multitude of computer vision tasks. While they are used to solve a variety of problems, the learning processes of CNNs are still not transparent. In recent years, many studies have been undertaken to explain these models [8,9]. However, the theoretical understanding of CNNs is still insufficient. In this paper, we use information theoretical measures to evaluate the learning process of CNNs. The research aims to answer three fundamental questions: How does the number of convolutional layers in the network affect the learning process in general? What are the effects of kernel size on network learning behaviour? What are the effects of hidden layers on the learning process of CNNs?

We are motivated by the studies on deep learning and information bottleneck theory by Tishby et al. [10,11,12]. In this study, mutual information across different layers of CNN is used to understand the effects of network parameters, such as kernel size, number of convolutional layers, dropout layer and dropout rate, on the learning process. Through this evaluation, we try to obtain some insights into the internal properties of CNNs by observing the mutual information between its interim layers. Thus, the effects of training on the learning process of a model are examined by observing the mutual information between the input and output of the model during training. We demonstrate that mutual information provides an indication of which layers are contributing to the learning of the features. The results further demonstrate that information theoretic tools can provide insight into the training procedure of the CNNs.

The rest of the paper is organised as follows: Section 2 reviews the related work in the understanding of deep learning and explainable machine learning. Section 3 describes the fundamentals of information theory utilised in this paper. The research methodology is provided in Section 4. It also explains the steps that are required in the information theory-based method to understand the learning processes of CNNs. In Section 5, the experimentation setups and results are discussed. Section 6 presents the conclusions and future work. 

## 2. Related Work

### 2.1. Understanding Deep Neural Networks

Deep learning, or so-called deep neural networks (DNNs), is a subfield of ML inspired by the structure and function of brain neurons. One of the advantages of deep learning over other types of ML is its scalable behaviour. Namely, the performance of deep learning models gets better as the amount of data used to train the model increases and features are automatically extracted by the network. The structure of DNNs consists of anything from several layers to millions of layers, which makes their mathematical explanation intractable. This nature of neural networks is thus somewhat “black box” in character [13].

Transparency which is aimed at a direct understanding of the model’s learning process can be said to be the opposite of the black box approach. When the neural network structure is considered, its input-output relations and model design can be expressed mathematically so that these properties can thus be defined as being transparent. However, layer parameters, the number of layers and non-linear properties are generally determined by traditional methods and heuristics. Since there is no optimisation of these parameters in a mathematical sense, the associated transparency is somewhat limited. In addition to these, because the selection of hyperparameters, such as the learning speed and batch size is also intuitive and has no transparent algorithmic structure, these networks are not reproducible. Thus, efforts [8,12] are being made to make DNNs more understandable and transparent.

Post-hoc explainability techniques are generally applied to deep learning models to explain their decisions. This method aims to understand how an already designed model (thus, it is also called post-modelling explainability) processes the information and gives the output [14]. These methods enhance the transparency of models that are not tractable in terms of explainable models, such as with deep neural networks. Some basic approaches to achieving this goal include visual explanations, local explanations and text explanations [15]. Zeiler and Fergus have tried to explain CNNs with a novel visualisation technique that gives information about intermediate layers [16]. Bae et al. have also introduced a textual explanation deep learning model for self-driving cars to obtain safe autonomous devices [17].

Besides computer science and statistical methods, some other techniques and procedures can be adopted to explain deep learning models. Information theory of communications systems has recently become one of the most referenced methods. The work of Ziv and Tishby, “Opening the Black Box of Deep Learning via Information” [10], as based on the Information Bottleneck Method [18], has led to a focus on explaining neural networks via information theoretic quantities such as mutual information. Other researchers have also investigated this [19,20]. Moreover, Yu et al. have introduced a new matrix-based Renyi’s α-entropy technique to analyse the information flow in stacked autoencoders and CNNs, respectively [9,21]. Furthermore, Balda et al. have adopted the information theoretic method and incorporated it with generalisation errors and suggesting the learning process of neural networks [12].

### 2.2. Explainable Machine Learning

Machine learning is a part of research into AI that aims to give computers the ability to learn, make and improve predictions based on data [22]. There are several types of ML algorithms depending on their learning style, i.e., supervised learning, unsupervised learning and semi-supervised learning. Nowadays, AI systems based on ML have been remarkably successful at various computer related tasks [4], from understanding natural languages [23] to playing complex games such as Go [24]. Most ML models such as deep neural networks are too complicated for people to understand easily due to their non-intuitive and opaque nature [25]. Hence, this lack of explainability of ML models acts as a barrier to the adoption of these models into application fields such as law, medicine and transportation. For example, knowing why a car performed a particular action is vitally important when designing self-driving cars [26]. As ML methods started to be used to make important predictions at critical points, the demand for transparency from the stakeholders of AI began to increase [27]. Thus, many researchers have been studying how to explain machine learning models recently. The aim of the research, which is generally called EML, is to help people understand and trust machine learning models in an intuitive manner.

EML is such a new and broad topic for the research community that it is yet to be formally defined. In [22] it is defined as the “science of understanding what a model did or might have done”, and in [28] it is thought as the “use of ML models for the extraction of relevant knowledge about domain relationships contained in data.” There is a range of reasons why some form of an explanation of a machine learning model is desirable. Adadi and Berrada reported that justifying decisions, enhancing control, improving models and discovering new knowledge are four fundamental reasons behind this desire [29]. To achieve this, some basic questions must be answered: How does the model work? Which inputs or features of the data are the most influential in determining output? What is the optimum mathematical representation of this model? Explainable models can be divided into three categories according to the purpose of motivating the associated research. These are explainability, interpretability and transparency. The first two are mostly about making a model, its internal process and its outputs intuitively humanly understandable. Transparency is about understanding the process of how the model or algorithm learns from the data [30]

If a model is self-explanatory, that model can be considered transparent. The transparency of a model can be measured in two different ways. Easily understandable models can be explained by methods during design. However, the explanations of some models are not at first tractable. For example, early machine learning models based on probabilistic mappings such as decision trees, logistic regression and clustering are convenient in terms of their explanations. Still, DNNs are very hard to understand [15], so recent research has begun to consider the learning behaviour of neural networks to make them more transparent [8,9]. Work towards understanding the learning procedure of CNNs, as described in this study, also contributes to the research in explainable machine learning.

## 3. Background

Information theory is concerned with quantification, storage and transfer of information. The quantification aspect of information theory, which is utilised in this paper, relates to measuring information related to distributions as based on probability and statistics. It was initially proposed and developed by Claude Shannon for communication system design [31,32,33]. This theory arises from the quest to determine how much information a signal contains.

### 3.1. Information Entropy

Information entropy or basically “entropy” is the measure of the uncertainty of a random variable that has a probability distribution. This quantity is described by the probability distribution, *p*(*x*), of the random variable *x*. Generally, entropy is an average quantity that depicts how much information an event or random variable contains.

**Definition** **1.** *Let X be a discrete random variable with a probability mass function*

p(x)=Pr{X=x}, x∈X

*the entropy H(X) of variable X is defined as*

(1) $$H(X)=-\sum_{x\in X}p(x)\log(x).$$

The unit of information entropy is measured in “bits” or “not” depends on the base of the logarithmic function being two or 𝑒, respectively.

### 3.2. Joint Entropy

In the case of two different random variables, the entropy of these values can be calculated in a similar manner to that of calculating entropy of a random variable. This term, called joint entropy, gives the overall uncertainty of the two random variables.

**Definition** **2.** *The joint entropy*  H(X,Y) *of a pair of discrete random variables, X and Y, with a joint distribution*  p(x,y) *is defined as*

(2) $$H(x,y)=-\sum_{x\in X}\sum_{y\in Y}p(x,y)\log p(x,y)$$

### 3.3. Conditional Entropy

Conditional entropy is a measure of the amount of information required to determine the outcome of a random variable *Y* given the value of the random variable of *X*.

**Definition** **3.** *The conditional entropy of Y given X, H(Y|X), is defined as:*

(3) $$H(Y|X)=-\sum_{x\in X}p(x)H(Y|X=x)$$

(4) $$=-\sum_{x\in X}p(x)\sum_{y\in Y}p(y|x)\log p(y|x)$$

(5) $$=-\sum_{x\in X}\sum_{y\in Y}p(y|x)\log p(y|x)$$

From the definitions of joint entropy and conditional entropy, it can be seen that the entropy of two random variables is the summation of the marginal entropy of one and the conditional entropy of the other. This theorem is called the chain rule of information entropy and is shown in (6). For the proof of the theorem, readers are referred to [33] (pp. 13–35).

**Theorem** **1.** *Chain rule*

(6) H(X, Y)=H(X)+H(Y|X)=H(Y)+H(X|Y)

### 3.4. Mutual Information

Mutual information describes the amount of information that one random variable contains about another.

**Definition** **4.** *For two random variables X and Y with a joint probability function*  p(x,y) *and marginal probability functions*  p(x) *and*  p(y) *, the mutual information*  I(X;Y) *is the relative entropy between the joint distribution and the product distribution*  p(x)p(y) *:*

(7) $$I(X;Y)=\sum_{x,y}p(x,y)\log\frac{p(x,y)}{p(x)p(y)}$$

This is the reduction in the uncertainty of one random variable due to the knowledge one has of the other. Thus, mutual information can be calculated [9] by entropy quantities as

(8) I(X; Y)=H(X)−H(X|Y)=H(Y)−H(Y|X)

**Figure 1:**
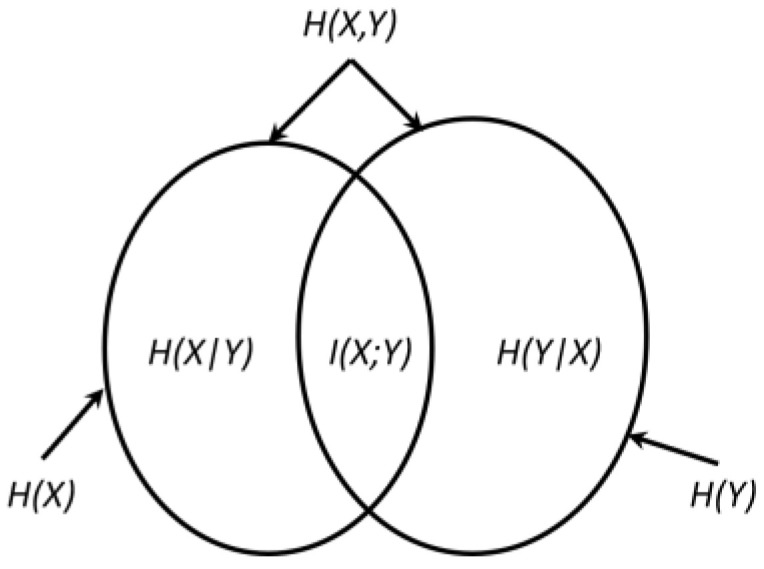
Relationship between entropy and mutual information.

From (7), it can be seen that the notion of mutual information is symmetric. Thus, *X* gives as much information about *Y* as *Y* gives about *X*. The relationship between these quantities is exhibited in Figure 1. From this figure, it can be understood that mutual information I(X; Y) is the intersection of the two random variables’ information.

### 3.5. Data Processing Inequalities

Data processing inequality (DPI) is an information theoretic concept that states that no physical processing of data in a Markov chain can increase its information content [9]. It can be said that the information that a variable contains cannot be increased by post-processing.

**Definition** **5.** *Random variables X, Y, Z are said to form a Markov chain in that order (denoted by*  X→Y→Z *) if the conditional distribution of Z depends only on Y and is conditionally independent of X.*

This results in the Data Processing Inequality theorem that no processing of Y, deterministic or random, can increase the information that Y contains about X [33] (pp. 34–35).

**Theorem** **2.** *Data processing inequality*

(9) If X→Y→Z, then I(X; Y) ≥ I(X; Z)

## 4. Methodology

In this section the evaluation methods that are used in the experiments are described. Then, the learning process for CNNs is investigated with information theoretic quantities that are presented in the previous sections.

### 4.1. Experimentation Methodology

The MNIST dataset [34] has been initially selected for evaluation in this study, and the Fashion-MNIST [35] dataset are used for the purpose of verification. The experiment consists of four main steps:

1. A convolutional neural network model is designed and compiled.
2. While the compiled model is being trained, the network weights are saved after every epoch.
3. The models selected for visualisation are rerun and the outputs of hidden layers (activations) are extracted and assigned to the predefined lists.
4. Finally, from these outputs, information quantities are calculated and plotted for evaluation.

**Figure 2:**
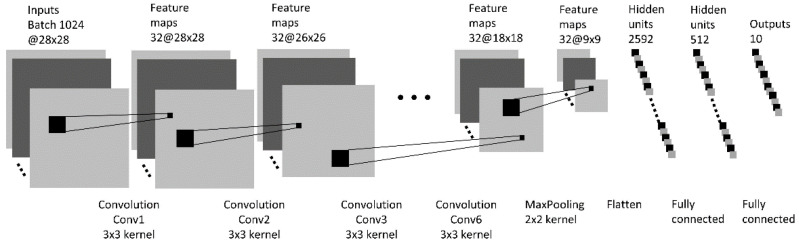
Neural network structure for the experiment. The network could have a maximum of six convolutional layers. The grey-scale input image contains 28 × 28 pixels.

The structure of the neural network used in the experiment is shown in Figure 2.

In order to evaluate the effect of mutual information between interim layers of a multi-layer neural network on the learning process, the neural network can be configured to include one to six convolutional layers. The last convolutional layer is followed by a max pooling layer, a flattening layer and two fully connected layers. All experiments were carried out with the Keras v2.3 Library [36] and the TensorFlow v2.3 backend [37]. The Python code for the experimentation is published in GitHub [38].

### 4.2. Datasets

The MNIST dataset is a modified subset of the National Institute of Standards and Technology database. This dataset contains 60,000 grey-scale images of handwritten digits represented by 28 × 28 pixels. The task is to classify a given picture of a handwritten digit into one of ten classes representing integer values from 0 to 9, inclusive. The Fashion-MNIST dataset is also used in this paper to compare the learning process with that of the MNIST dataset. The Fashion-MNIST dataset is based on clothing items and also contains 60,000 grey-scale images of 28 × 28 pixels. There are 10 clothing class labels including T-shirt, trousers, pullover, etc., and the classification task is to classify a given picture of item into one of the 10 clothing classes. For each dataset, we use 50,000 images for network training and 10,000 images for validation.

### 4.3. Calculation of Information Quantities

In this study, information theoretic quantities are used to understand the behaviour of CNNs. To calculate these quantities, all representations of the input samples and the output of each layer are considered to be individual random variables. Then, for each experiment, the mutual information I(X;T) between the input *X* and output *T* of the investigated layers are calculated empirically by (7) and (8). The computing algorithm can be described as follows:

Step 1: The input image matrices or output tensors, and the investigated layer’s output, are converted to one-dimensional arrays.

Step 2: Each unique value in the previously generated arrays and the occurring frequencies of those values are counted. Then, from these counts, the occurrence probability of each value is calculated.

Step 3: From the probabilities obtained in Step 2, the entropy and mutual information of variables are computed by using (1), (7) and (8).

### 4.4. Information Quantities of the MNIST Images

**Figure 3:**
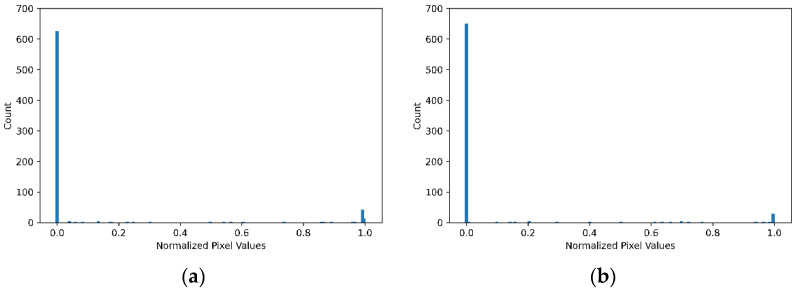
Histogram of normalised pixel values of two randomly selected images from the MNIST dataset. (**a**) Pixel value histogram of Image 1 (image sequence number 150 in the dataset); (**b**) Pixel value histogram of Image 2 (image sequence number160 in the dataset).

**Figure 4:**
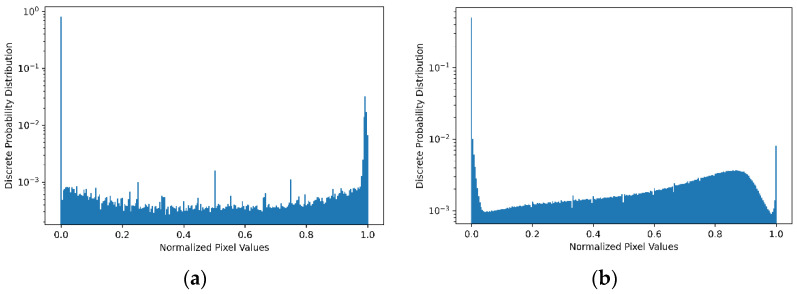
Discrete distributions of image pixel values in two datasets. (**a**) Distribution in the MNIST dataset; (**b**) Distribution in the Fashion-MNIST dataset. Note that the probability is shown in logarithmic scale.

The discrete pixel values of images in the MNIST dataset are normalised from 0–255 to 0.0–1.0 for easy processing of data. The pixel value histograms of two randomly selected images are shown in Figure 3 for the purpose of observing the general pixel structure in the dataset. The discrete distribution of normalised pixel values for the entirety of the MNIST and Fashion-MNIST training datasets are shown in Figure 4, which illustrates the probability of a pixel value appears in a selected image in the dataset. Because of the grey-scale nature of the dataset, Figure 3 and Figure 4a shows the MNIST pixel values are concentrated around 0.0 and 1.0. This indicates that in the MNIST images, most pixels are either brighter (represented by pixel value of 255) or darker (represented by a pixel value of 0). It is also noticeable, in Figure 4b, although the Fashion-MNIST pixel values are also concentrated around darker value 0, they are less concentrated around the brighter value 255 but more evenly distributed across all other values. This is an indication that classification of the Fashion-MNIST dataset is more difficult than that of the MNIST dataset.

From the probability of pixel value information, the theoretical information quantities such as entropy, mutual information and conditional entropy are calculated empirically. The calculated theoretic information quantities of the two randomly selected MNIST images are shown in Table 1.

It can be seen from the histograms of the selected images shown in Figure 3 that the distributions of pixel values are approximately the same. Further, it can be inferred that the amount of uncertainty is relatively low due to the black and white structure of the images. Thus, the amount of entropy was expected to be low. The fact that the entropy values meet expectations gives us some assurance about the appropriateness of the calculation method. Moreover, it can be seen from Table 1 that the mutual information is the subtraction of the marginal conditional entropy from the dominant entropy, which demonstrates the compatibility of the method in (8).

## 5. Experiments and Results

This section contains the results of the proposed methodology experimentation conducted to answer our three main research questions with information-theoretic quantities:

- How does the number of layers in the network affect the learning progress?
- What are the effects of kernel size on CNN network’s learning behaviour?
- What are the effects of dropout layer on the learning of CNN networks?

The learning behaviour of the convolutional neural networks was evaluated using mutual information during the training of the classification tasks. In this study representations of input and output of layers are vectorised and considered as random variables. The input and output of each layer, and the true labels of the dataset are denoted by *X*, *T* and *Y* hereafter, respectively. During the experimentation, the mutual information, I(X;T), between the input and output of the layers was calculated. As mentioned in Section 4.3, the input and output of each layer in the network were considered as individual variables and the mutual information between the model’s input and output, between each layer’s input and output and between the model’s output and the true labels were calculated from those variables.

### 5.1. Experiment 1—Effect of Number of Convolutional Layers on Learning

**Figure 5:**
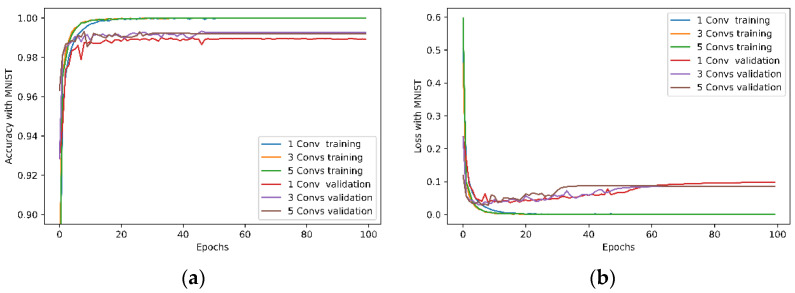

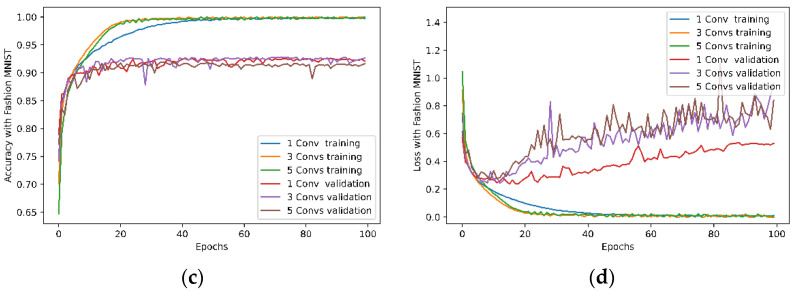
The accuracy and loss curves in training and validation with different convolutional layers and kernel size 3 × 3. (**a**) Accuracy with the MNIST dataset; (**b**) Loss with the MNIST dataset; (**c**) Accuracy with the Fashion MNIST dataset; and (**d**) Loss with the Fashion MNIST dataset. Note for readability reasons, only curves of 1, 3 and 5 convolutional layers are shown.

**Figure 6:**
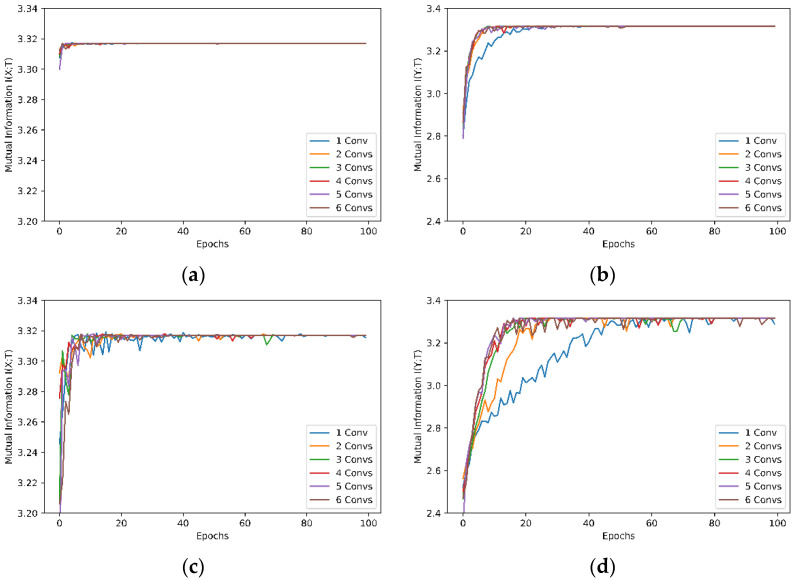
Mutual information of six neural network models with kernel size 3 × 3. (**a**,**c**) Mutual information *I(X;T)* between the models’ input and output with the MNIST and Fashion-MNIST datasets, respectively; (**b**,**d**) Mutual information *I(Y;T)* between the models’ output and true labels with the MNIST and Fashion-MNIST datasets, respectively.

**Figure 7:**
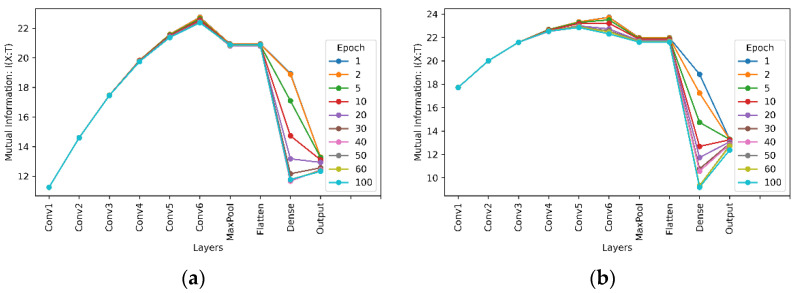
Mutual information between interim layers of a neural network with six convolutional layers and kernel size 3 × 3. (**a**) For the MNIST dataset; (**b**) For the Fashion-MNIST dataset.

To examine the effect of the number of convolutional layers on learning, six neural network models with varying numbers of convolutional layers were trained. The remaining part of the models, including the max pooling layer, the flatten layer and the fully connected layers were not changed. All network models have the same structure as shown in Figure 2. After training, the accuracy and loss data in training and validation were first collected and shown in Figure 5, and the following information quantities were then calculated and plotted:

- Mutual information between the model’s input and output, I(X;T), as shown in Figure 6a,c;
- Mutual information between the model’s output and true labels, I(Y;T), as shown in Figure 6b,d;
- Mutual information between the input and output of interim layers through the network with six convolutional layers for the MNIST and Fashion-MNIST datasets, as shown in Figure 7.

### 5.2. Experiment 2—Effect of Kernel Size of Convolutional Layer on Learning

**Figure 8:**
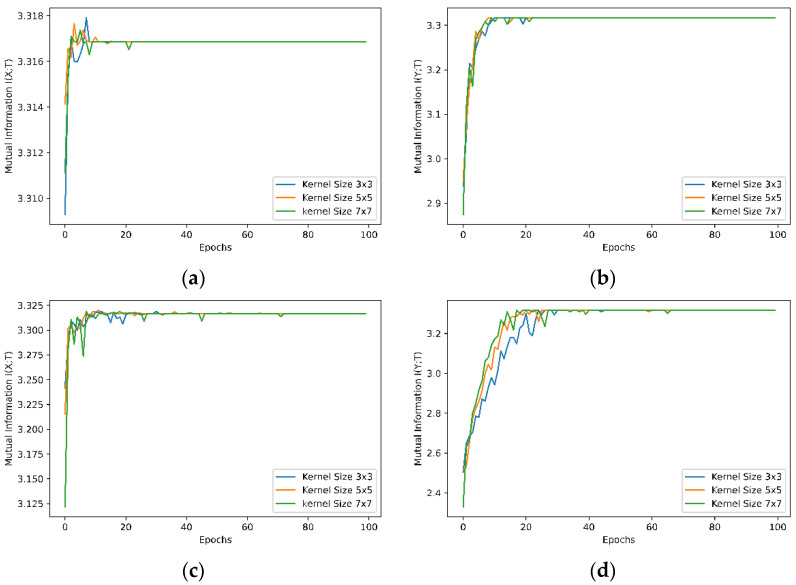
Mutual information with convolutional layer kernel size 3 × 3, 5 × 5 and 7 × 7. (**a**,**c**) The mutual information *I(X;T)* between the network input and output for the MNIST and Fashion-MNIST datasets, respectively; (**b**,**d**) The mutual information *I(Y;T)* between the network output and the true labels for the MNIST and Fashion-MNIST datasets, respectively.

**Figure 9:**
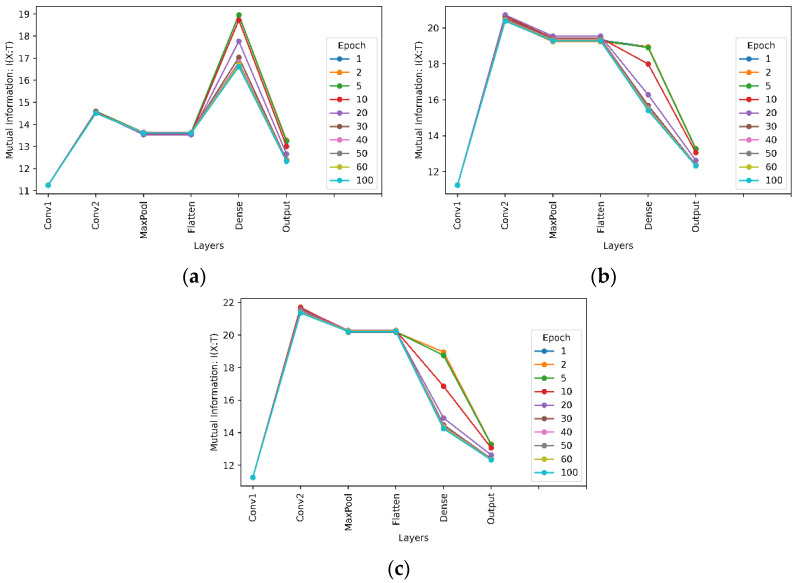
Mutual information between interim layers of three neural networks with different convolutional layer kernel sizes for the MNIST dataset. (**a**) Kernel size 3 × 3; (**b**) Kernel size 5 × 5; (**c**) Kernel size 7 × 7.

**Figure A1:**
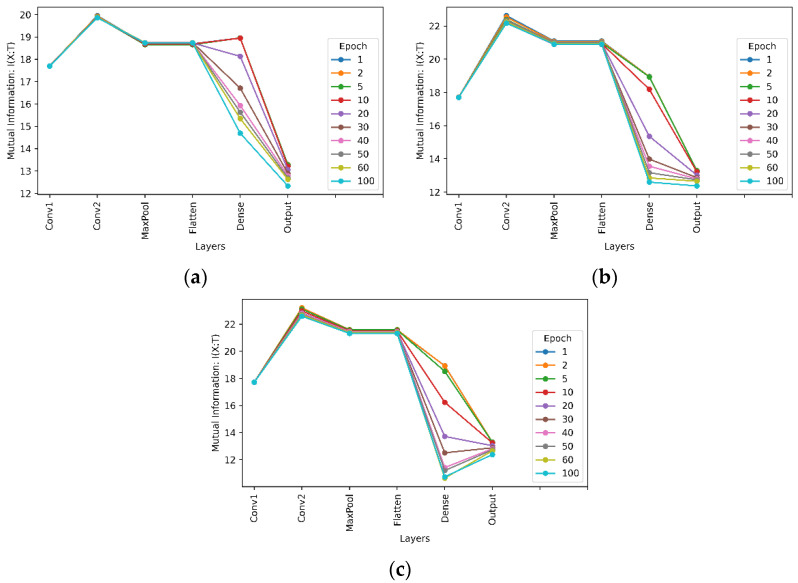
Mutual information between interim layers of three neural networks with different convolutional layer kernel sizes for the Fashion-MNIST dataset. (**a**) Kernel size 3 × 3; (**b**) Kernel size 5 × 5; (**c**) Kernel size 7 × 7.

To examine the effect of kernel size of convolutional layers on learning, three different neural networks have been trained. The three networks have the same structure (as shown in Figure 2) with two convolutional layers. The kernel sizes of the three networks are 3 × 3, 5 × 5 and 7 × 7, respectively. We compare the mutual information I(X;T) between the network input and output, mutual information I(Y;T) between the output and true labels for both the MNIST and Fashion-MNIST in Figure 8, and the mutual information between a network’s interim layers for the three networks for the MNIST dataset in Figure 9. The results of mutual information between a network’s interim layers with different kernel sizes for the Fashion-MNIST dataset are shown in Figure A1 in the Appendix A.

### 5.3. Experiment 3—Effect of Dropout Layer on Learning

**Figure 10:**
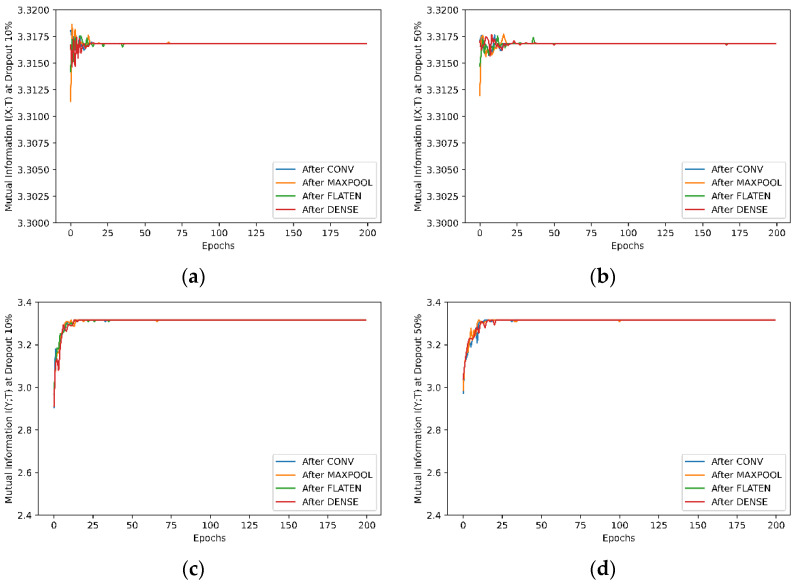
Mutual Information when a dropout layer is applied to different hidden layers for the MNIST dataset. (**a**,**b**) Mutual information *I(X;T)* between input and output with dropout rate at 10% and 50%, respectively; (**c**,**d**) Mutual information between output and true labels with dropout rate at 10% and 50%, respectively. The mutual information with different dropout layer position for the Fashion-MNIST dataset is shown in Figure A2 in the Appendix A.

**Figure A2:**
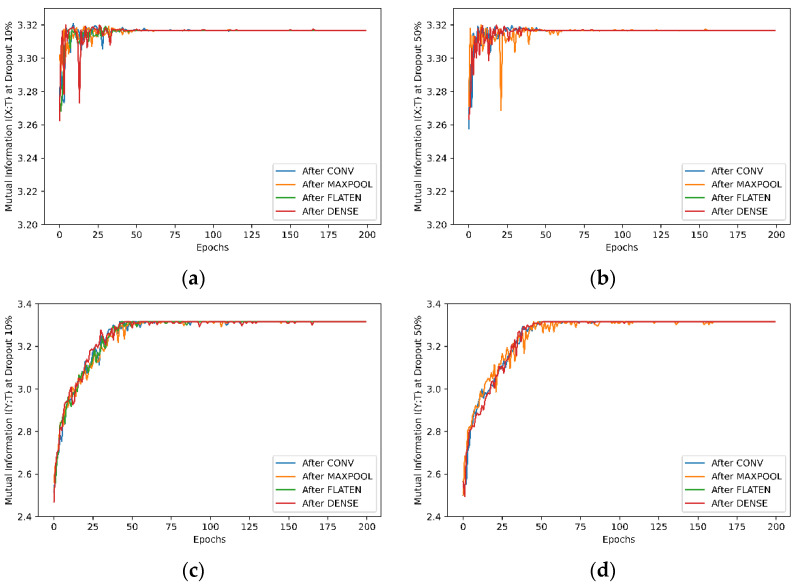
Mutual Information when a dropout layer is applied to different hidden layers for the Fashion-MNIST dataset. (**a**,**b**) Mutual information *I(X;T)* between input and output with dropout rate at 10% and 50%, respectively; (**c**,**d**) Mutual information *I(Y;T)* between output and true labels with dropout rate at 10% and 50%, respectively.

**Figure A3:**
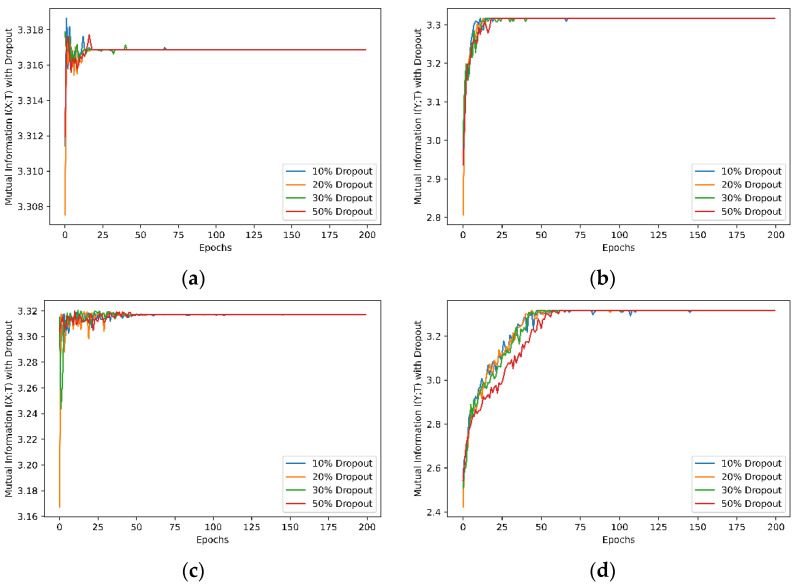
Mutual information with a dropout layer at different dropout rates applied to max pooling layer. (**a**) Mutual information *I(X;T)* between the network input and output with the MNIST dataset; (**b**) Mutual information *I(Y;T)* between the network output and the true labels with the MNIST dataset; (**c**) Mutual information *I(X;T)* with the Fashion-MNIST; (**d**) Mutual information *I(Y;T)* with the Fashion-MNIST.

Mutual information is also used to evaluate the learning of neural networks with a dropout layer being applied to different layers in the network. The dropout layer randomly sets input units to 0 with a specified dropout rate at each step during training time. By observing the changes of mutual information with a dropout layer being applied to different network layers, we could get more insights into the learnings of the network. In this experiment, the same network structure as shown in Figure 2 is used with two convolutional layers. The dropout layer is applied separately after the convolution layers, max pooling layer, flattening layer and fully connected dense layer. In each case, the mutual information is collected with dropout rate 0.1, 0.2, 0.3 and 0.5. For the MNIST dataset, the results with the dropout layer at rates of 10% and 50% being applied at different hidden layers are illustrated in Figure 10. The results of dropout rates at 10% and 50% for the Fashion-MNIST are shown in Figure A2 in the Appendix A. As the dropout layer is commonly used after the max pooling layer in machine learning, we have extracted the results of different dropout rates after the max pooling layer and have shown them in Figure A3 in the Appendix A.

### 5.4. Main Findings

#### 5.4.1. Effects of Number of Convolutional Layers on Learning

As shown in Figure 5a,c, the models can achieve a validation accuracy of above 99% with the MNIST dataset and 90% with the Fashion-MNIST dataset with a varying number of convolutional layers. The figure also shows that a higher number of convolutional layers results in better accuracy, but there are no apparent gains in validation accuracy when there are more than three convolutional layers with the MNIST dataset. However, we cannot draw the same conclusion when the Fashion-MNIST is used in the same model. The results of training and validation loss, shown in Figure 5b,d, suggest that the networks start overfitting after around 10–15 epochs for both the MNIST and Fashion-MNIST datasets.

The effects of the number of convolutional layers on mutual information between the network input and output and between output and the true labels are shown in Figure 6. As shown in Figure 6a,c, the mutual information I(X;T) between the input and output for networks with different numbers of convolutional layers is only slightly different at the start of training, and it converges to a stable value after around 10 (for the MNIST dataset) or 20 (for the Fashion-MNIST) epochs. However, there is no significant difference in I(X;T) with different numbers of convolutional layers in the network. The mutual information I(Y;T) between the network output and the true labels for the MNIST dataset, as shown in Figure 6b, converges to a stable value more quickly than the I(Y;T) of the Fashion-MNIST dataset shown in Figure 6d. This proves the assumption that the Fashion-MNIST is a more challenging machine learning task than the MNIST dataset. More importantly, mutual information approaching a stable maximum quantity with sufficient epochs in training is an indication that the network is not able to learn more information from the dataset even with more training epochs. The maximal mutual information in Figure 6 is 3.3168, which is less than the theoretical maximum mutual information value of 3.3219 for 10 labels, reflecting the fact that the training and validation accuracy is less than 100%.

Figure 6b,d also indicate that there is no significant difference in mutual information I(Y;T) with more than two or three convolutional layers in the network for the MNIST and Fashion-MNIST datasets, respectively. This could potentially be used as an indicator in optimising neural network configuration.

In the context of this study, the mutual information between the input *X* and the output *T* of hidden layers is calculated by (10).

**Theorem** **3.** *Information sharing*

(10) If variable T is a deterministic function of X, then I(X; T)=H(T)

This assumption was made because it is known that deep learning models are deterministic [39]. Based on this assumption and (10), the effect of hidden layers on learning is evaluated and the results are shown in Figure 7 for a network with six convolutional layers for the MNIST and Fashion-MNIST datasets. It shows how the information-theoretic quantities have changed between the hidden layers.

From the results of mutual information between the interim layers of the network, as shown in Figure 7, it is clear that convolutional layers consistently increase the amount of mutual information, or the entropy according to (10), between interim layers. Figure 7 also shows that the max pooling layer reduces the mutual information. This is because the max pooling layer reduces the dimensionality of its output by reducing the number of pixels in the output from its previous convolutional layer. With no changes in mutual information after the flattening layer, we can conclude that the flattening layer has no effect on learning, as the flattening layer merely flattens the input to a one-dimensional vector and no information will be lost during this linear operation. Noticing that for interim layers from the first convolutional layer to the flatten layer, there is nearly no difference in the entropy between different training epochs. However, there are significant decreases of entropy with more training epochs in the dense layer, meaning that more training epochs reduce the uncertainty after the dense layer. It was thus inferred that most learning takes place in the fully connected dense layers, which fits the definition of a CNN. In a CNN, convolutional blocks are used to extract features from the image. The fully connected layers are added to make the classification. The mutual information between interim layers with the Fashion-MNIST dataset shows similar behaviour.

#### 5.4.2. Effect of Kernel Size of Convolutional Layers on Leaning

Figure 8 shows that changing the kernel size of convolutional layers does not change the learning behaviour of the neural network significantly in the case where only two consecutive convolutional layers are used sequentially. In all three cases with kernel size 3 × 3, 5 × 5 and 7 × 7, the values of mutual information are very close to each other, although the mutual information I(Y; T) between output and true labels of kernel size 3 × 3 with the Fashion-MNIST, shown in Figure 8d, is reaching the same value as that of kernel size 5 × 5 and 7 × 7 a few epochs later in training. However, when the training time of the models with different kernel sizes was considered, as shown in Table 2, it is clear that the time spent for learning increases with larger kernel sizes, and the small kernel sizes give good balance between learning performance and computation time required.

Figure 9 shows the effect of different kernel sizes on the learning of interim layers with two convolutional layers in the network for the MNIST dataset. It is obvious, as shown in Figure 9a, that the convolutional layers with kernel size 3 × 3 increase mutual information less than the convolutional layers with kernel size 5 × 5 and 7 × 7 (Figure 9b,c). The larger the kernel size, the greater the mutual information between the convolutional layers. This could be interpreted as indicating that convolutional layers with larger kernel size get more spatial information from images than convolutional layers with smaller kernel size during learning. The behaviour of other interim layers, including the flatten layer and fully connected dense layer, is not affected by the kernel size. With the Fashion-MNIST dataset, the effect of different kernel sizes on the learning of interim layers, as shown in Figure A1 in the Appendix A, is similar to the MNIST dataset.

#### 5.4.3. Effect of Dropout Layer on Learning

The dropout layer’s effect on neural network learning is show in Figure 10, and Figure A2 and Figure A3 in the Appendix A. It is indicated, in Figure 10 and Figure A2, that given the same dropout rate, the dropout layer’s places do not have as much of an effect on the learning because similar mutual information between the network input and output and between the output and true labels for differently placed dropout layers has been observed.

When the dropout layer is only applied to the max pooling layer, Figure A3 in the Appendix A shows the changes of mutual information between network input-output and output-true labels with different dropout rates. A general observation from Figure A3 is that the dropout rates do not affect the input-output mutual information much in learning. However, a higher dropout rate at 50% does reduce the mutual information between the output and true labels and slow down the mutual information converging to its stable value with the Fashion-MNIST dataset. This is due to that fact that a higher dropout rate means more input units of the dropout layer are set to zeros randomly, and therefore more random changes are introduced to the learning than in a lower dropout rate, resulting in lower mutual information between the output and the true labels. In training, the learning is also noticeably slower with higher dropout rates.

The convolutional neural network could also be investigated regarding whether it satisfies DPI or otherwise. In the case of a feed-forward neural network, the Markovian structure and data processing inequalities across layers are generally accepted [10,12]. In a previous study [9], it was stated that this could also be seen in CNN networks, despite the calculation limits. However, in tests carried out with the proposed approach in this study, there was no investigation on DPI between the layers for CNN networks. Hence, the investigation of data processing inequality theory for CNN networks could be conducted in future.

### 5.5. Future Work

The proposed method can benefit from further development, especially to improve the practicality of applying it in complex settings. First, all the information quantities mentioned in this paper are calculated by taking all variables in one-dimensional vectors, i.e. the input images or output of any layer is first converted to a single vector before entropy or mutual information of the variables are calculated. While this calculation is straightforward, it could result in the spatial relationships in the image-related data being ignored. Therefore, the question remains as to whether the information theoretic estimation is reliable, and whether that estimation is feasible within a tensor structure. In future studies, developing a calculation method by considering this spatial relation will give more reliable results. Second, the results presented in this paper are from a series of prognostic tests. While this can reveal the empirical relations between mutual information and parameters of the neural networks, it does not necessarily explain the underlying learning mechanisms directly. Mutual information alone (as opposed to e.g., the rate-distortion theory) is incapable of capturing information associated with the accuracy of the estimates as it is essentially sensitive to correlations between stochastic processes and not point-wise discrepancies between the underlying probability measures. In addition to this, further useful investigations, as listed as follows, could be conducted to investigate the learning of CNNs based on the information theoretical measures:

(1) By applying different numbers of inputs to the training model, the effect of the number of training data on learning could be observed with the same approach.
(2) By using different optimisation functions for the model during the learning process, the effect of the optimisation functions on learning could be observed.
(3) The reliability of the proposed method could be determined by trying different estimation methods for mutual information calculation.

## 6. Conclusions

The main aim of this work was to understand the learning process of CNNs by utilising information theoretic measures in a series of prognostic tests. As a result of the study, the effects of hidden layers and training process on neural network learning could be observed and stated.

The MNIST and Fashion-MNIST datasets were used in training different model setups. Based on the experiments and the analysis of the results, we found that more than two or three convolutional layers did not necessarily improve the learning of CNNs with the MNIST and Fashion-MNIST datasets, although the exact number of layers may vary depending on the specific classification task. Larger kernel sizes do not improve the learning of convolutional layers significantly. With the dropout rate in the range of 10% to 50% in our experiments, where the dropout layer is applied to has no obvious effects on learning. For dropout rates as high as 50%, the results indicate that when the dropout layer is applied to the max pooling layer, it could reduce the mutual information between the network output and true labels and reduce the speed at which the mutual information converges to its stable value in learning.

## Figures and Tables

**Table 1:** Information quantities for the two randomly selected MNIST images.

Type of Information Quantities	Quantity
Entropy of Image 1, *H(X)*	1.5731
Entropy of Image 2, *H(Y)*	1.5342
Mutual Information, *I(X;Y)*	1.4191
Conditional Entropy, *H(X|Y)*	0.1540
Conditional Entropy, *H(Y|X)*	0.1151
Joint Entropy, *H(X, Y)*	1.6882

**Table 2:** Training results of networks for different kernel size with two convolutional layers with the MNIST dataset.

Kernel Size	Training Time Per Epoch (ms)	Validation Loss	Validation Accuracy
3 × 3	1026	0.0549	0.9920
5 × 5	2029	0.0493	0.9927
7 × 7	2031	0.0366	0.9931
9 × 9	3047	0.0666	0.9933

## Data Availability

The data presented in this study are openly available in GitHub [38].
